# Rethinking osteocyte models *in vitro*: the need for mineralised extracellular matrix and network architecture

**DOI:** 10.3389/fbioe.2026.1895189

**Published:** 2026-08-27

**Authors:** Małgorzata Morenc, Flavia Medeiros Savi, Alexandra Tits, Pascal R. Buenzli, Richard Weinkamer, Peter Fratzl, Nathalie Bock

**Affiliations:** 1 Max Planck Queensland Centre for the Materials Science of Extracellular Matrices (MPQC), Brisbane, QLD, Australia; 2 School of Biomedical Sciences, Faculty of Health, Queensland University of Technology (QUT) at the Translational Research Institute (TRI), Centre for Biomedical Technologies (CBT), Brisbane, QLD, Australia; 3 School of Mechanical, Medical and Process Engineering (MMPE), Faculty of Engineering, (QUT), Centre for Biomedical Technologies (CBT), Brisbane, QLD, Australia; 4 Max Planck Queensland Centre for the Materials Science of Extracellular Matrices (MPQC), Potsdam, Germany; 5 Department of Biomaterials, Max Planck Institute of Colloids and Interfaces, Potsdam, Germany; 6 School of Mathematical Sciences, Faculty of Science, Queensland University of Technology (QUT), Brisbane, QLD, Australia; 7 ARC Industrial Transformation Training Centre (ITTC) for Microphysiological System Technology (MiPSET), Queensland University of Technology, Brisbane, QLD, Australia

**Keywords:** bone tissue engineering, bone-on-chip, lacunocanalicular network, mechanobiology, microphysiological systems, mineralised extracellular matrix, osteocyte network, osteocyte models

## Abstract

Osteocytes inhabit one of the most extreme cellular microenvironments in the human body: a hydrated, hierarchically structured mineralised extracellular matrix where lacunar confinement, canalicular connectivity, fluid transport and mechanics are inseparable from cell function. *In vitro* osteocyte models have rapidly advanced through 3D culture, bioprinting, patterning, microfluidics, multicellular systems and controlled loading. Yet physiological interpretation often remains limited when marker expression, dendrite formation or bulk mineral deposition are assessed without defining the matrix and network architecture that deliver local cues. Here, we review engineered osteocyte microenvironment models through a matrix-centred and network architecture lens. We highlight advances in biomimetic mineralisation, viscoelastic and patterned hydrogels, dynamic microphysiological platforms and ECM-cell characterisation. We propose that progress will depend on modular, cross-correlative and computationally usable models that connect mineralised ECM, osteocyte network topology, transport and mechanics. Such models could move the field from descriptive osteocyte-like cultures toward interpretable niches that explain osteocyte behaviour.

## 1 Introduction

Osteocyte modelling has advanced rapidly, but the criteria used to define a physiologically informative model remain incompletely defined. *In vitro* osteocyte systems now include 3D printed hydrogels (Yang et al., 2020; Zhang J. et al., 2022), human cells (Zhang J. et al., 2022; Knowles et al., 2023; Woo et al., 2025; Zauchner et al., 2024), controlled patterning (Zauchner et al., 2024; Gehre et al., 2024; Bernero et al., 2024; Müller et al., 2025), microfluidic and bone-on-chip platforms (Woo et al., 2025; Yvanoff and Willaert, 2022; Poudel et al., 2025; Merife et al., 2025), bioreactors (Zhang J. et al., 2022; Merife et al., 2025; Rindt et al., 2024), and biomimetic mineralisation (Thrivikranam et al., 2019; De Wildt et al., 2026). Validation, however, still often emphasises osteocyte marker expression and dendrite-like morphology, whereas the matrix and fluid transport features that define the native niche are less incorporated. This emphasis was a necessary starting point for establishing osteocyte-like models *in vitro*, but the next step is to develop more integrated criteria for physiological relevance.

Osteocyte identity *in vitro* is usually assessed through combined morphological, molecular and functional readouts, which may include dendrite formation, E11/podoplanin, DMP1, PHEX/MEPE, SOST/sclerostin and FGF23 expression, connexin 43-mediated communication, RANKL/OPG regulation and responses to mechanical stimulation (Choi et al., 2022; Robling and Bonewald, 2020). These readouts have been explored using murine osteocyte-like cell lines, especially MLO-Y4 and IDG-SW3 (Yang et al., 2020; Bernero et al., 2024; Yvanoff and Willaert, 2022; Poudel et al., 2025; Merife et al., 2025), primary human osteocytes or osteoblast-to-osteocyte differentiation models (Knowles et al., 2023; Woo et al., 2025; Skottke et al., 2019; Wirsig et al., 2024; Wirsig et al., 2022; Bernhardt et al., 2021; Nasello et al., 2020), and hMSC-derived osteogenic constructs (Zhang J. et al., 2022; Zauchner et al., 2024; Thrivikranam et al., 2019; De Wildt et al., 2026). However, because osteocyte-derived readouts are strongly shaped by the surrounding matrix, they are best interpreted in relation to mineralised ECM architecture.

Osteocytes are embedded within a hierarchically structured mineralised matrix whose orientation is reflected in the osteocyte network itself (Kerschnitzki et al., 2011). Bock et al. argued that extracellular matrix should be treated as a material whose functions arise from multiscale organisation, water organisation, fluid transport properties, mechanics and adaptation, rather than primarily from biochemical composition (Bock et al., 2024). Bone exemplifies this principle. Its functions depend on mineralised collagen fibrils, non-collagenous proteins, water, canalicular transport, lacunar confinement and cell-mediated remodelling (Bock et al., 2024; Fritton and Weinbaum, 2009; Ayoubi et al., 2021). The osteocyte lacunocanalicular network is embedded within a highly organised mineralised matrix (Ayoubi et al., 2021; Roschger et al., 2019) and functions as a reciprocal cell–matrix–fluid system: its architecture shapes fluid flow and mechanosensation (Wang et al., 2007), while osteocyte mechanosignalling regulates bone modelling, remodelling and network adaptation (Lerebours and Buenzli, 2016; Wang et al., 2022; Mehrpooya et al., 2026).

Mineralisation in *in vitro* models therefore needs to be defined beyond its presence or absence. Reznikov et al. framed ECM mineralisation as a regulated materials process involving calcium and phosphate homeostasis, amorphous precursors, collagen architecture, mineral inhibitors and promoters, and spatial gradients (Reznikov et al., 2016). In biofabricated models, bulk calcium deposition or the addition of hydroxyapatite particles will not reproduce the mineralised bone ECM. For mineralisation to be physiologically interpretable, it is useful to report not only whether mineral is present, but also its composition, spatial distribution and relationship to the surrounding matrix and cells (Choi et al., 2022; van Tol et al., 2020).

Recent studies show both progress and challenges. Gehre et al. used photosensitised two-photon ablation to carve micron-scale channel grids into GelMA hydrogels, guiding hMSC protrusion outgrowth and 3D cell network formation with user-defined architecture (Gehre et al., 2024). Poudel et al. used femtosecond laser-assisted cavitation to generate user-defined 3D ‘Cellnets’ in collagen, including MLO-Y4 networks with defined connectivity and real-time Ca^2+^ signal propagation under flow and biochemical stimulation (Poudel et al., 2025). Merife et al. developed a microfluidic platform in which 3D MLO-Y4 collagen networks were exposed to unidirectional flow for up to 21 days, combining calcium imaging, connexin 43 inhibition, gene expression and matrix deformation assessment (Merife et al., 2025). Woo et al. built a human BMU-on-chip triculture that coordinated osteocyte, osteoblast and osteoclast precursor interactions across remodelling-like phases (Woo et al., 2025). Together, these studies represent meaningful progress. They also clarify what remains difficult. Some models incorporate mineralisation but do not yet capture osteocyte network architecture (Thrivikranam et al., 2019). Others improve cellular patterning and network function but do not yet incorporate mineralised ECM (Poudel et al., 2025). Dynamic systems now use flow, loading and multicellular interactions, but the local stimulus experienced by osteocytes is shaped by the collagen or hydrogel matrix through which those cues are transmitted (Woo et al., 2025; Merife et al., 2025). These differences should therefore be framed as design choices: first, by defining which niche feature each model is built to reproduce, and second, by asking how these features shape mechanistic conclusions about osteocyte biology.

**FIGURE 1:**
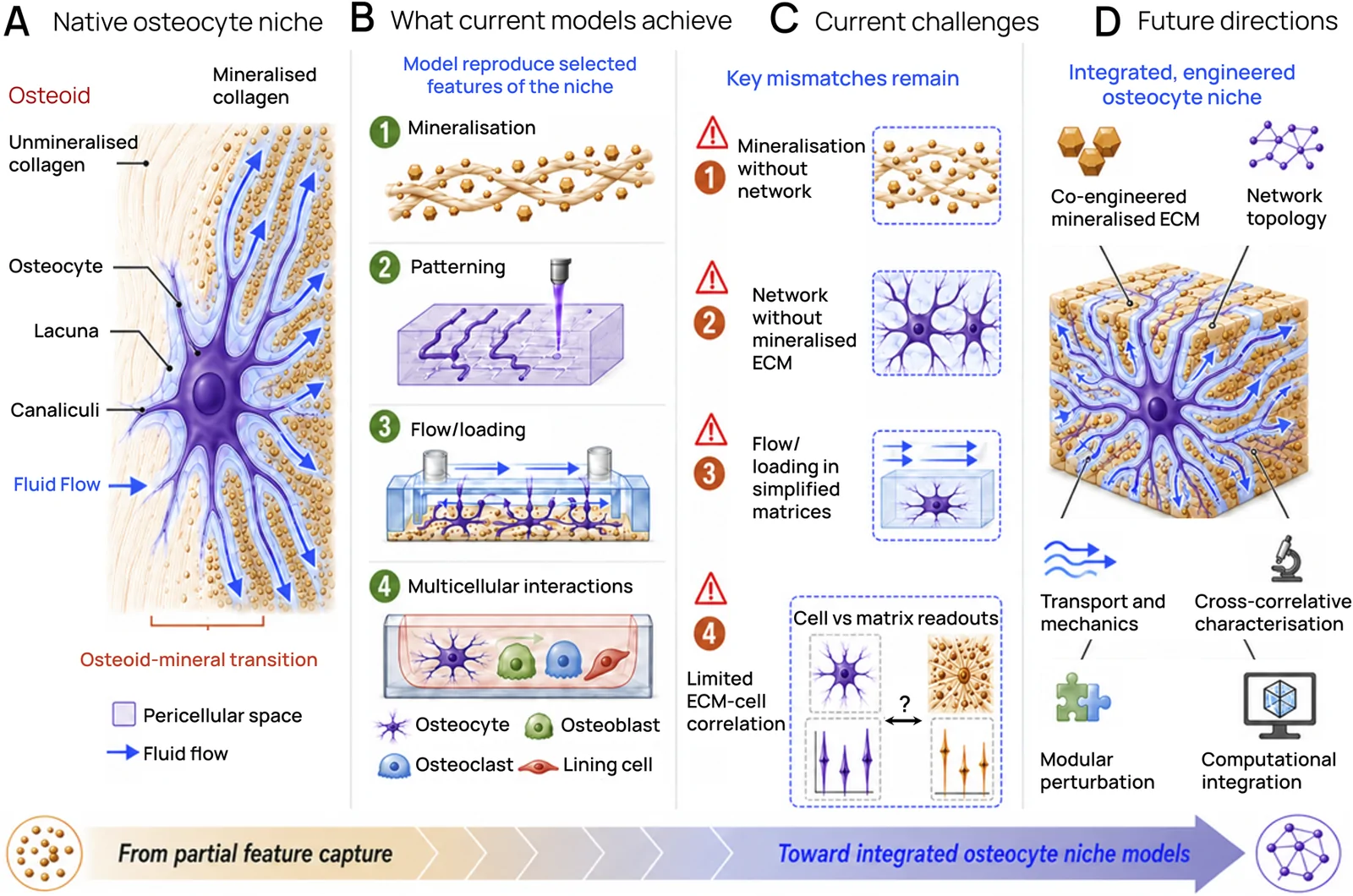
Conceptual framework for osteocyte microenvironment modelling. **(A)** The native osteocyte niche is a coupled mineralised collagen, lacunar, canalicular, pericellular and fluid-transport network. **(B)** Current models reproduce selected niche features, including mineralisation, patterning, flow or loading, and multicellular interactions. **(C)** Current challenges remain where mineralisation, network topology, dynamic stimulation and ECM-cell characterisation are developed separately. **(D)** Future advances will benefit from co-engineering mineralised ECM and osteocyte network architecture, while incorporating transport and mechanics, modular perturbation, cross-correlative characterisation and computational integration.

**FIGURE 2:**
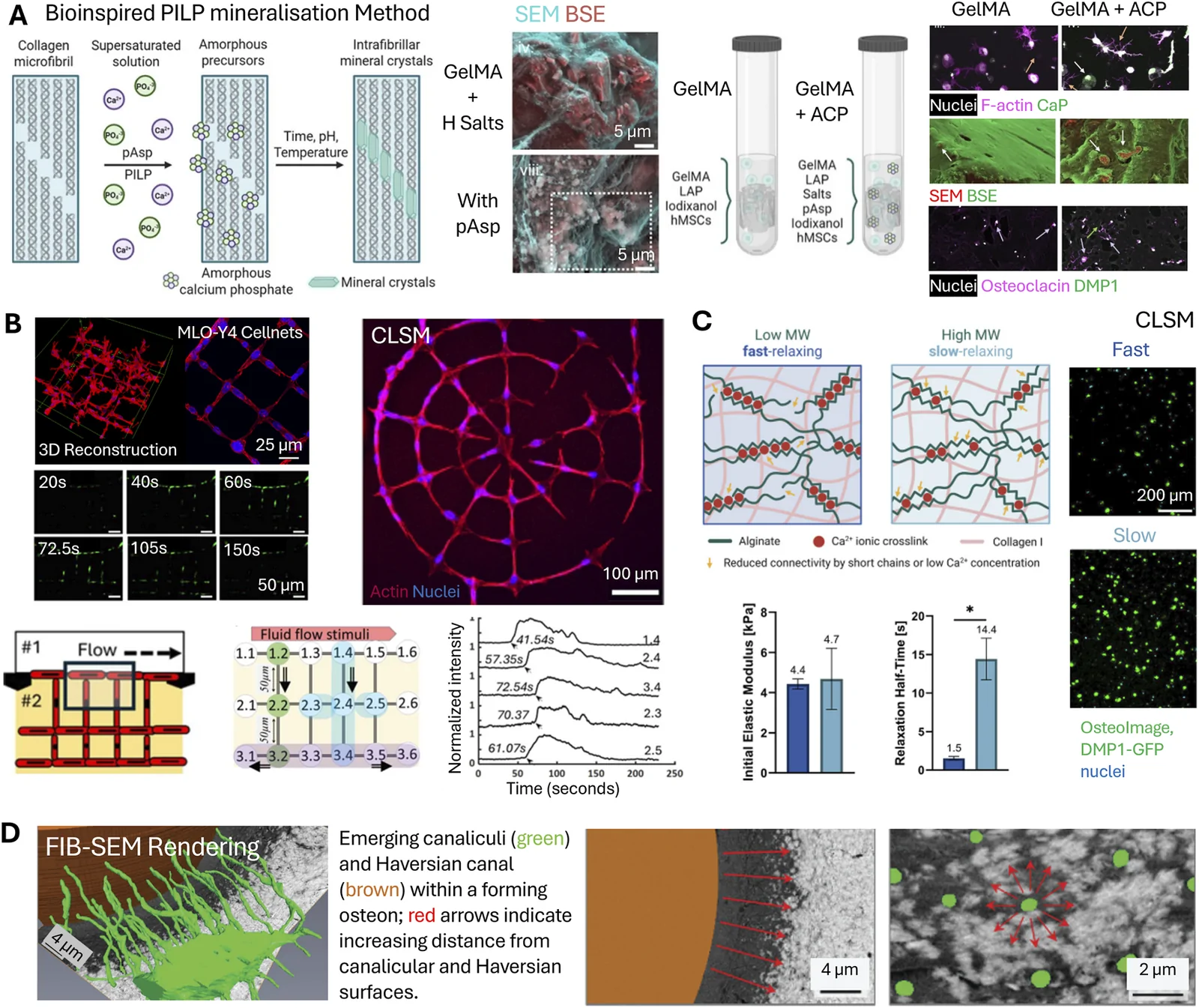
Recent experimental **(A–C)** and characterisation **(D)** advances that inform engineered osteocyte niche design. **(A)** Development of a mineralising gelatin methacryloyl (GelMA) bioresin by combining polymer-induced liquid-phase precursor (PILP) mineralisation with refractive-index tuning for volumetric bioprinting (VBP). Poly-aspartic acid (pAsp) stabilised amorphous calcium phosphate (ACP) precursors, improving light transmission and enabling trabecular-like, high-density human mesenchymal stromal cell (hMSC)-laden constructs. Mineralising resins were characterised by scanning electron microscopy/backscattered electron detection (SEM/BSE), Fourier-transform infrared spectroscopy (FTIR) and energy-dispersive X-ray spectroscopy (EDX), while printed constructs maintained viability, showed mineral-associated staining and expressed early osteocytic markers, including osteocalcin and dentin matrix acidic phosphoprotein 1 (DMP1) (De Wildt et al., 2026) (LAP: lithium phenyl-2,4,6-trimethylbenzoylphosphinate, H salts: high concentration salts (27 mM CaCl_2_ 12.6 mM K_2_HPO_4_), CaP: Calcium Phosphate). **(B)** Femtosecond laser-assisted cavitation to generate user-defined 3D ‘Cellnets’ in collagen. Concentric ring architectures illustrate controlled network patterning, while MLO-Y4 ‘Cellnets’ enabled real-time Ca^2+^ signal propagation under flow stimulation (Poudel et al., 2025). **(C)** Bernero et al. engineered alginate-collagen hydrogels with similar stiffness but distinct stress relaxation, showing that matrix viscoelasticity can regulate osteocyte morphogenesis, DMP1-green fluorescent protein reporter signal (DMP1-GFP) and mineral-associated signals (Bernero et al., 2024) (CLSM: Confocal Laser Scanning Microscopy). **(D)** Focused ion beam scanning electron microscopy (FIB-SEM) was used to map the three-dimensional relationship between osteocyte lacunocanalicular architecture and forming mineral in human bone, highlighting the spatial coupling between mineralisation fronts and canalicular structures (Ayoubi et al., 2021). Panels reproduced or adapted under publisher licences.

Here, we review engineered osteocyte microenvironment models through a matrix-centred and network architecture lens. We discuss key challenges in the field (Figure 1), consider how matrix, network and functional characterisation can strengthen physiological interpretation, and highlight recent experimental and analytical advances (Figure 2).

## 2 The osteocyte niche as a mineralised cell-matrix network

The osteocyte niche is best understood as a coupled material and transport network. Its critical features are not simply that the matrix is stiff, mineralised or 3D, but that mineral, collagen, lacunae, canaliculi, pericellular matrix and fluid pathways are organised across multiple length scales (Fritton and Weinbaum, 2009; van Tol et al., 2020; Reznikov et al., 2014). These features determine how external mechanical and biochemical cues are redistributed before they reach the cell.

Mineral content affects both mechanics and fluid transport. Mineralisation generally increases stiffness but reduces toughness when excessive (Reznikov et al., 2016). At the ultrastructural scale, deformation is partitioned across collagen, mineral and interfacial proteins, so tissue-level strain is not transmitted uniformly to fibrils or mineral crystals (Gupta et al., 2006). At the microscale, lacunae, canaliculi and pores generate local strain gradients and fluid-flow-related stimuli around osteocytes (van Tol et al., 2020; Weinbaum et al., 1994; Knothe Tate, 2003). Mineral deposition can also alter permeability and solute exchange; for example, subcanalicular nanochannel volume is inversely correlated with calcium content in human cortical bone (Tang et al., 2023). For engineered models, mineral supplementation may therefore change not only stiffness, but also hydration, pore connectivity and transport pathways.

Collagen organisation provides another level of control. Collagen fibrils in lamellar bone are not mineralised randomly; they show preferred orientation, with local disorder at interfaces and around osteocyte spaces (Tits et al., 2023). This contributes to crack deflection and energy dissipation, and also shapes the osteocyte network (Wagermaier et al., 2015). Osteocyte network orientation mirrors ECM orientation (Repp et al., 2017a), and network architecture correlates with bone material quality (Kerschnitzki et al., 2013). Thus, dendrite orientation is not only a cell-intrinsic phenotype; it is also influenced by the surrounding matrix architecture. Dendrites formed in isotropic gels may reflect osteocyte-like morphology without necessarily reflecting native-like matrix guidance.

Because bone is exceptionally stiff, osteocyte stimulation depends strongly on amplification through the lacunocanalicular system. The Fluid Flow Hypothesis proposed that loading-induced fluid movement generates shear stress on osteocytes (Fritton and Weinbaum, 2009; Weinbaum et al., 1994; Knothe Tate, 2003). Solute movement depends on LCN pore structure and loading (Wang, 2018), and bone mechanoresponse is closely related to LCN architecture rather than simply to applied strain magnitude (van Tol et al., 2020). Osteocyte network architecture may also contribute to a material embodiment of the mechanical setpoint (Lerebours and Buenzli, 2016). Thus, the same external load can generate different local stimuli depending on network geometry.

For *in vitro* models, the practical implication is that osteocyte behaviour is best interpreted in relation to the matrix architecture that delivers the stimulus. Rather than requiring every platform to reproduce the full mineralised lacunocanalicular niche, each model can be defined by the features it captures, such as mineral distribution, transport properties, matrix orientation, network connectivity and mechanical signal transmission. This provides a framework for matching model design to the biological question being asked.

## 3 Static systems

Static 3D systems have produced some of the most useful osteocyte-like phenotypes because they allow long culture times, matrix deposition and controlled biomaterial design. Their interpretation, however, depends strongly on how well the matrix context is defined.

### 3.1 Non-mineralised collagen and hydrogel models

Collagen gels remain widely used because collagen I is the dominant organic matrix component of bone and supports cell adhesion, dendritic morphology and mineral deposition. Bernhardt and colleagues developed primary human osteocyte networks in collagen and later expanded them into coculture and triculture models with osteoblasts and osteoclasts, showing lineage-associated gene expression and cell-type-specific enzyme activities, including ALP in osteoblasts and TRAP, cathepsin K and carbonic anhydrase II in osteoclasts (Skottke et al., 2019; Wirsig et al., 2024; Wirsig et al., 2022; Bernhardt et al., 2021). The triculture model is particularly useful for paracrine signalling (Bernhardt et al., 2021), although its interpretation remains within an unmineralised matrix context.

Nasello et al. showed that primary human osteoblast differentiation in collagen was cell density-dependent: high-density cultures developed longer protrusions, increased ALP, downregulated BSP2 and expressed DMP1 by 21 days (Nasello et al., 2020). This highlights cell density and 3D confinement as important regulators of osteoblast-to-osteocyte transition. Gelatin- and alginate-based hydrogels provide additional control over matrix mechanics. Bernero et al. recently used alginate-collagen interpenetrating networks with comparable stiffness but different stress relaxation times (Figure 2C) (Bernero et al., 2024). Fast-relaxing gels promoted early spreading and longer processes, whereas slow-relaxing gels favoured later ALP, DMP1, hydroxyapatite signal and gap junction connectivity. This is important because it separates stiffness from viscoelasticity and suggests that matrix relaxation can tune osteocyte morphogenesis and network formation (Bernero et al., 2024; Nguyen et al., 2021).

### 3.2 Mineralised hydrogel and bioprinted platforms

Hydrogel platforms commonly introduce mineralisation through induction media or calcium/phosphate supplementation, allowing mineral deposition to be studied within cell-laden, tuneable matrices.

Yang et al. demonstrated that bioprinting can impose spatial control on osteocyte-like cultures (Yang et al., 2020). IDG-SW3 cells were printed in Gelatin Methacryloyl/Hyaluronic Acid Methacryloyl (GelMA/HAMA) hydrogels with or without collagen I, then cultured in mineralisation induction medium. Collagen-containing constructs showed stronger osteocyte-like behaviour, including increased DMP1-GFP signal, calcium deposition, E11/Podoplanin expression and dendrite formation (Yang et al., 2020). These are concrete achievements, but mineralisation was still mainly assessed through cell-associated calcium deposition and bulk hydrogel outcomes, leaving nanoscale or intrafibrillar mineral organisation as an important target for future specialised characterisation.

Da Costa Sousa et al. mineralised GelMA microgels using CaCl_2_, osteopontin and K_2_HPO_4_, confirming apatite-like minerals by FTIR, EDX and TEM, while maintaining osteocyte viability (da Costa Sousa et al., 2024). However, dendrite length and number were not significantly improved in mineralised microgels, supporting that mineralisation alone does not establish a mature osteocyte network.

Together, these studies show that mineralisation can be incorporated into hydrogel-based osteocyte models, but also highlight the need to distinguish mineral deposition within a hydrogel from organised mineralised collagen architecture that develops during osteocyte embedment in bone matrix.

### 3.3 Biomimetic and matrix-centred mineralisation

Here, biomimetic mineralisation is used in a matrix-centred sense: not simply the presence of a mineral, but deliberate control of mineral precursor chemistry or collagen-associated mineral deposition and matrix maturation.

Thrivikraman et al. embedded hMSCs in type I collagen hydrogels and used a supersaturated calcium and phosphate medium stabilised with milk-derived osteopontin to direct nanoscale apatite deposition into intra- and extrafibrillar collagen spaces (Thrivikranam et al., 2019). The matrix was characterised by EDX, TEM, FTIR, SEM, SAED, and AFM. Mineralisation increased the modulus of individual hydrated collagen fibrils from approximately 0.0016 GPa–2.21 GPa. The system promoted osteogenic and pre-osteocytic features in hMSCs, including osteocalcin, DMP1 and E11/Podoplanin expression, dendrite-like cellular projections extending through the mineralised matrix and lacuna-like collagen-free regions around embedded cells. Although this is an important contribution, one remaining limitation is that SOST gene expression and sclerostin protein were not reported, and the phenotype was framed mainly as osteoblastic or pre-osteocytic (Thrivikranam et al., 2019).

De Wildt et al. recently extended biomimetic mineralisation into volumetric biofabrication by combining PILP mineralisation with refractive-index tuning (Figure 2A) (De Wildt et al., 2026). pAsp-stabilised amorphous calcium phosphate precursors enabled optically printable GelMA-based bioresins and resulting trabecular-like constructs with high-density hMSC encapsulation. The mineralising resin was characterised by SEM/BSE, FTIR and EDX, while optical-density and light-scattering analyses linked precursor stabilisation to improved printability. The resulting constructs showed high viability, increased calcium deposition under osteogenic conditions and early osteocytic features, including dendrite-like morphology and expression of podoplanin, osteocalcin and DMP1. Importantly, the authors also noted that direct *in situ* characterisation of mineral phase remains challenging, reinforcing the need for specialised and cross-correlative mineral characterisation (De Wildt et al., 2026).

Iordachescu et al. approached the challenge of coupling osteocyte-like network formation with mineralised matrix maturation through self-organisation rather than predefined matrix design (Iordachescu et al., 2018). A fibrin gel cast between calcium phosphate ceramic anchors supported periosteal cells to deposit an ordered collagen-mineral matrix over long-term culture. Raman spectroscopy and XRD confirmed hydroxyapatite associated with collagen, SHG showed collagen organisation comparable to mature mouse femora, and nano-computed tomography revealed canaliculi-like connections between osteocyte-like cells. This model is valuable because matrix maturation, mineralisation and osteocyte-like embedding emerge together, although prolonged culture and lower architectural control limit its practicality compared with more reductionist engineered platforms (Iordachescu et al., 2018).

### 3.4 Patterning and confinement

Patterned models attempt to control network architecture by guiding cell spacing, alignment and connectivity. Early microbead-guided perfusion systems showed that physical scaffold architecture can steer 3D osteocyte network formation, with human osteoblast-derived cells forming dendritic networks aligned with the microbeads under microfluidic perfusion (Gu et al., 2015; Sun et al., 2017). Zhang et al. later compared non-confined MLO-Y4 monolayers with cells confined under GelMA or mineralised GelMA, enabling real-time calcium imaging and showing spatially heterogeneous Ca^2+^ responses across connected osteocyte networks, indicating that confinement changes how flow-induced signals propagate (Zhang K. et al., 2022).

Gehre et al. used photosensitised two-photon ablation to carve micron-scale channels into GelMA hydrogels containing hMSCs (Gehre et al., 2024). Addition of the P2CK photosensitiser reduced the ablation threshold five-fold, enabling low-energy patterning of 1 µm wide, 30 µm deep channels; after 7 days, cells extended protrusions into the channels and formed grid-aligned 3D networks (Gehre et al., 2024). This is an elegant yet technically demanding example of user-defined canaliculi-like guidance.

Poudel et al. advanced this architectural direction using femtosecond laser-assisted cavitation to create user-defined 3D microchannel networks in native collagen within microfluidic chips (Figure 2B) (Poudel et al., 2025). MLO-Y4 cells formed viable ‘Cellnets’ with defined connectivity, real-time Ca^2+^ signal propagation under flow and ionomycin stimulation, and redirected signalling after targeted network injury (Poudel et al., 2025). Although non-mineralised, this strongly links 3D network architecture to functional perturbation.

Microporous hydrogels generated by photopolymerisation-induced phase separation provide a complementary route to predefined laser channels. Recent PEG hydrogels with 5–20 µm pores and PVA-dextran sulphate hydrogels generated by PIPS with 2–40 µm pores supported rapid 3D spreading, dendritic morphogenesis, osteogenic readouts, microfluidic integration and volumetric printing (Zauchner et al., 2024; Müller et al., 2025), offering useful routes for transport-permissive, cell-guiding hydrogel architectures.

Scaffold-scale topographical studies, such as those by Dhimmar et al. and Soheilmoghaddam et al., further indicate that geometric and chemo-topographical cues can support osteogenic alignment, mineral deposition and osteocyte-associated marker expression, while highlighting the need to better connect these outcomes with organised mineralised ECM (Dhimmar et al., 2024; Soheilmoghaddam et al., 2024).

## 4 Dynamic and microphysiological systems

Dynamic osteocyte models introduce fluid flow, mechanical loading, spatial gradients, multicellular interfaces and time-dependent cues that static cultures cannot easily capture. Their interpretation, however, depends on distinguishing the stimulus applied to the platform from the stimulus experienced by osteocytes within the engineered matrix.

Microfluidic systems are particularly useful for controlling osteocyte communication. Yvanoff and Willaert used robotic cell printing to create spatially defined osteocyte-osteoblast arrays, enabling analysis of connexin 43-mediated communication, calcium waves, AFM-based mechanical stimulation and fluid-flow responses (Yvanoff and Willaert, 2022). This approach provides precise control over heterotypic cell-cell communication under dynamic conditions. Merife et al. extended microfluidic stimulation to self-assembled MLO-Y4 networks in collagen under pulsatile unidirectional fluid flow stimuli (Merife et al., 2025). Daily pulsatile flow triggered Ca^2+^ signalling across 3D osteocyte networks, maintained several osteocyte-associated genes for up to 21 days, and enabled estimation of collagen deformation, stress and velocity fields during stimulation (Merife et al., 2025). These studies provide useful dynamic platforms, while integration of mineralised ECM architecture remains an important next step.

Perfusion and mechanical loading bioreactors expose osteocyte-like cells to controlled 3D mechanical environments (Rindt et al., 2024; Wilmoth et al., 2020). Rindt et al. developed a bioreactor with independent flow and cyclic compression using IDG-SW3 cells on decellularised bone, laser-textured mineralised collagen and β-TCP scaffolds (Rindt et al., 2024). Laser-textured mineralised collagen supported homogeneous repopulation and dynamic differentiation; perfusion increased DMP1, SOST and CX43, while compression upregulated mechanosensitive genes including FOS and PTGS2 (Rindt et al., 2024). This provides a strong example of controlled 3D mechanostimulation.

Human BMU-on-chip systems extend dynamic modelling toward coordinated remodelling. Woo et al. differentiated primary human osteoblasts into osteocytes within collagen gels, then introduced osteoblasts and osteoclast precursors to model phases of the bone remodelling cycle (Woo et al., 2025). Compared with transwell culture, the chip increased osteoblast- and osteocyte-associated responses, remodelling-related signalling and osteoclast-associated activity (Woo et al., 2025). This provides a useful platform for spatiotemporal cell-cell signalling, while mineral-enriched ECM and vascular components remain future opportunities.

Dynamic platforms are also relevant for disease modelling and drug screening because bone diseases and fracture repair involve coupled changes in cell-matrix properties (Mansoorifar et al., 2021; Lin et al., 2022). For matrix-dependent questions, these systems should report not only flow, compression or chip geometry, but how those cues are transmitted through the ECM. Bessot et al. support this principle: mineralisation-supplemented osteogenic preculture promoted more lamellar-like collagen, improved osteocyte alignment and generated regions with higher local elastic modulus, despite limited changes in osteocyte density or dendrite number (Bessot et al., 2025). This reinforces a useful design principle for *in vitro* models: where possible, matrix composition should be linked to network organisation and local mechanics, rather than assessed separately.

Dynamic systems should therefore be viewed as complementary platforms. The next challenge is to connect their strengths with better-defined mineralised matrix architecture.

## 5 Characterising ECM-cell coupling

Future advances in *vitro* osteocyte modelling may come less from adding more complex devices than from coupling biofabrication with rigorous characterisation of mineral, matrix, transport and network architecture. Bock et al. argue for multiscale, multimodal and time-resolved ECM analysis, with attention to specimen preparation, hydration, transport and structural coordinates (Bock et al., 2024).

Because mineralisation is intricately linked with network architecture and transport processes, it is important that relevant matrix characteristics are reported, such as mineral amount, its spatial distribution, as well as collagen organisation, hydration and permeability alongside mechanical properties. Raman spectroscopy can define ECM chemistry, mineral-to-matrix signatures and, when polarised approaches are used, aspects of collagen organisation, and has been applied in mineralised bone-like constructs (Iordachescu et al., 2018; Bergholt et al., 2019). FTIR and XRD help confirm mineral phase, crystallinity and mineral-to-matrix ratios (Thrivikranam et al., 2019; Iordachescu et al., 2018). SEM/EDX, qBEI and micro-computed tomography map mineral distribution and volume (Thrivikranam et al., 2019; Paris et al., 2017; Repp et al., 2017b). SAXS/WAXS can then resolve mineral particle thickness and orientation, with SAXS linking collagen alignment to mineral orientation across a soft-to-mineralised interface (Paris et al., 2017). TEM and SAED localise intra- and extrafibrillar mineral (Thrivikranam et al., 2019), while FIB-SEM provides 3D nanoscale information on the relationship between mineralisation and the lacunocanalicular network (Figure 2D) (Ayoubi et al., 2021). SHG and CLSM are particularly useful for correlating collagen organisation with osteocyte network alignment (Repp et al., 2017a), while nanoindentation and rheology connect architecture to local or bulk mechanics (Bernero et al., 2024; Bessot et al., 2025).

Alizarin red and von Kossa staining are useful screening readouts, but they do not show mineral phase, collagen association or spatial distribution relative to cells and network spaces. Follow-up methods should therefore be chosen to test the relevant mechanism, such as mineral chemistry, mineral distribution or collagen–mineral organisation. Bessot et al. provide a useful example by adapting TIMA, a tool commonly used for geological mineral analysis, to bioengineered bone (Bessot et al., 2025). By correlating collagen-I and collagen-II immunohistochemistry with BSE and TIMA-EDX maps, they linked matrix identity to mineral composition, showing distinct Ca/P ratios in collagen-I-positive mineralised bone and collagen-II-positive mineralising cartilage (Bessot et al., 2025). This type of workflow shifts mineralisation assessment from asking whether mineral is present to asking what mineral is present, where it is located, and how it relates to matrix identity and organisation.

Network characterisation should also move beyond representative images. Yang et al. quantified cell body volume, dendrite number and dendrite length in bioprinted IDG-SW3 constructs using Image-Pro Plus 6.0, alongside CX43, E11, DMP1, mineral deposition and PTH response (Yang et al., 2020). Bernero et al. used IMARIS to quantify osteocyte area and dendrite length in viscoelastic alginate-collagen hydrogels, then related morphology to stress relaxation, ALP, DMP1, hydroxyapatite and gap junction formation (Bernero et al., 2024). Future studies could gain further insight by mapping these metrics against mineral density, collagen organisation, local stiffness and transport pathways. Because tools such as IMARIS and Image-Pro Plus are costly, validated open-source segmentation workflows will be increasingly important, alongside specialised tools such as TINA for quantifying osteocyte lacunocanalicular network architecture (Repp et al., 2017b).

Functional characterisation remains essential. DMP1, E11, SOST, FGF23 and CX43 are useful markers, but should be paired where possible with PTH responsiveness (Yang et al., 2020), RANKL/OPG regulation, calcium signalling, flow response, mechanosensitive genes and effects on osteoblasts or osteoclasts (Woo et al., 2025). The strongest future studies will not simply show more complex networks; they will use cross-correlative workflows to register live cell imaging, collagen organisation, mineral chemistry, mechanics and transport in spatially matched datasets.

In practice, the methods described in this section need not all be applied in every study; as a core standard, osteocyte viability and phenotype should be combined with 3D confocal imaging and quantitative network analysis using established general-purpose software (e.g., Imaris), while LCN-specific tools such as TINA (Repp et al., 2017b) represent a more advanced option requiring specialised expertise. Where mechanics or transport are investigated, accessible first-line methods include bulk rheology or compression testing and fluorescent-tracer diffusion or FRAP, respectively, while nanoindentation and spatially resolved transport mapping represent advanced approaches (Bock et al., 2024). Polarised Raman mapping, micro-CT/qBEI and SHG form a further advanced tier, whereas SAXS/WAXS, TEM/SAED and FIB-SEM are specialist, resource-intensive methods, and their integration into fully registered correlative workflows remains aspirational for many laboratories.

## 6 Perspective

Reproducing the osteocyte niche *in vitro* is intrinsically difficult because it spans multiple length scales, from tissue-level mechanics to nanoscale mineral and fluid transport, while coupling matrix architecture, cellular networks and biochemical signalling. The main limitation of current osteocyte models is therefore not simply lack of complexity, but incomplete coupling between these features. Cell-centred systems can generate osteocyte-like markers, dendrites and signalling responses (Yang et al., 2020; Bernero et al., 2024; Skottke et al., 2019; Wirsig et al., 2024; Wirsig et al., 2022; Bernhardt et al., 2021; Nasello et al., 2020; Bernhardt et al., 2020; Bernhardt et al., 2019), while matrix-centred systems can reproduce aspects of mineralised collagen, tissue organisation and local mechanics (Thrivikranam et al., 2019; Iordachescu et al., 2018; Bessot et al., 2025; Paris et al., 2017). The next challenge is to connect these strengths into models where mineralised ECM, network architecture, transport and mechanics can be interpreted together.

Furthermore, although recreating physiologically relevant osteocyte microenvironments remains a broad ambition, physiological fidelity should not be treated as a universal or all-encompassing design goal, because the features that must be reproduced depend on the biological question. The matrix is both an instructive *input* and a cell-generated, dynamically remodelled *output*; therefore, the choice between reproducing a mature niche architecture and recreating the processes by which it emerges should also be question-dependent. Two complementary modelling principles can be distinguished. Engineered reconstruction places osteocytes within matrices of predefined composition, mechanics, mineralisation or LCN-like geometry and is therefore well suited to reproducible mechanistic comparisons and pharmaceutical screening (Yang et al., 2020; Bernero et al., 2024; Zhang K. et al., 2022). By contrast, self-organising, emergent models can draw on transferable principles of native niche formation: osteoblasts organise and deposit collagen-rich osteoid, a subset becomes embedded and extends dendritic processes, and mineralisation progresses in close spatial association with the developing LCN (Ayoubi et al., 2021). This sequence can be exploited using osteoblast-lineage cells at appropriate densities (Nasello et al., 2020), permissive cell-remodellable matrices (Zauchner et al., 2024), and spatial or mechanical boundary cues that allow matrix deposition, cellular embedment, network formation and mineralisation to evolve together (Zhang J. et al., 2022; Iordachescu et al., 2018). Where reciprocal cell–matrix interactions are central, principles of self-organisation can also be incorporated into engineered reconstruction through hybrid models that define the initial matrix composition, microporosity and boundary conditions while permitting cell-mediated matrix degradation and deposition, network formation and progressive mineralisation (Zauchner et al., 2024; Gehre et al., 2024).

A modular strategy may offer the clearest route forward. Increasing complexity is not automatically beneficial, because adding several variables at once can obscure causal interpretation. Instead, well-defined modules, such as mineralised matrices, patterned networks, mechanical stimulation, multicellular signalling or remodelling-like interfaces, can be validated independently and then progressively combined. This approach would allow each platform to be matched to the biological question being asked: a microfluidic chip may be most useful for cell-cell signalling, a bioreactor for controlled mechanical stimulation, and a self-organising construct for matrix maturation.

Cell source remains part of this design problem. MLO-Y4 is useful for dendritic, gap-junction-connected networks and live mechanosignalling, but has limited matrix-mineralising and late endocrine capacity (Merife et al., 2025). Although less represented in engineered 3D models, OCY454 has been explored in 3D hydrogel/scaffold systems (Nguyen et al., 2021; Hoppock et al., 2023), and offers more mature osteocyte signalling than MLO-Y4, including robust DMP1-reporter and SOST/sclerostin-related responses, making it useful for studying osteocyte-like signalling in mineralising matrix environments. Conversely, IDG-SW3 supports osteoblast-to-osteocyte differentiation (Yang et al., 2020; Rindt et al., 2024). Primary human osteoblast/osteocyte models improve human relevance but are less scalable (Knowles et al., 2023; Nasello et al., 2020). hMSC- or periosteal-cell systems are strong for engineered matrix deposition, mineralisation and early osteocytic embedding, but mature osteocyte function requires further validation (Thrivikranam et al., 2019; De Wildt et al., 2026; Iordachescu et al., 2018). These differences should be treated as design choices rather than limitations in themselves (Creecy et al., 2021).

Mathematical and computational models can complement *in vitro* systems by linking measurable architecture to predicted function. Models of fluid flow in the LCN, signal propagation and osteocyte network growth already show that network architecture influences permeability, mechanosensitivity and cell embedding dynamics (Lerebours and Buenzli, 2016; Mehrpooya et al., 2026; Buenzli, 2015; Taylor-King et al., 2020; Mehrpooya et al., 2024). Multiscale models link loading, scaffold architecture, tissue formation and remodelling (García-Aznar et al., 2021; Perier-Metz et al., 2020). However, these approaches remain difficult to validate *in vivo* and have not yet been widely applied to biofabricated osteocyte systems. Controlled *in vitro* platforms with defined matrix and network perturbations could therefore provide valuable datasets for refining and validating computational models (Figure 1D).

This strategy requires reporting parameters that are both biologically meaningful and computationally usable, including collagen organisation, mineral phase and distribution, Ca/P ratio, stiffness, viscoelasticity, permeability, lacuna-like confinement, dendrite length, branching and connectivity. Conversely, simulations could help guide experimental design by predicting which matrix architectures, pore sizes, mineral gradients or flow regimes are most likely to generate physiologically relevant osteocyte stimulation. Related modelling work at soft tissue-to-bone interfaces similarly shows that simulations can reveal load-transfer strategies that are difficult to infer experimentally alone (Tits and Ruffoni, 2021). This would be especially valuable for disease modelling and drug screening, where changes in cell behaviour, matrix organisation and mechanics are coupled (Mansoorifar et al., 2021; Lin et al., 2022; Bessot et al., 2025).

Overall, progress in osteocyte modelling will depend on moving beyond phenotype mimicry toward predictive niche engineering. The aim is not to fully replicate bone *in vitro*, but to reproduce the niche features most relevant to a specific biological question. For this selective approach to be mechanistically useful, platforms may need to define the matrix and network features they are designed to test, co-engineer osteocyte networks and mineralised ECM where these features are central to the question (Figure 1D), and characterise them through spatially matched matrix, mechanical and functional readouts that can inform computational models. This shift will move the field from descriptive osteocyte-like cultures toward models that can explain how mineralised network architecture regulates osteocyte behaviour.

## Glossary

3D: three-dimensional
ACP: amorphous calcium phosphate
AFM: atomic force microscopy
ALP: alkaline phosphatase
BMU: basic multicellular unit
BSE: backscattered electron microscopy
BSP2: bone sialoprotein 2
Ca/P: calcium-to-phosphate ratio
CLSM: confocal laser scanning microscopy
COL1A1: collagen type I alpha 1 chain
CX43: connexin 43
DMP1: dentin matrix acidic phosphoprotein 1
ECM: extracellular matrix
EDX: energy-dispersive X-ray spectroscopy
FGF23: fibroblast growth factor 23
FIB-SEM: focused ion beam scanning electron microscopy
FTIR: Fourier-transform infrared spectroscopy
GelMA: gelatin methacryloyl
GFP: green fluorescent protein
HAMA: hyaluronic acid methacryloyl
hMSC: human mesenchymal stem cell
IDG-SW3: osteocyte-like cell line
IL-6: interleukin-6
LCN: lacunocanalicular network
MEPE: matrix extracellular phosphoglycoprotein
MLO-Y4: murine long-bone osteocyte Y4 cell line
MMP: matrix metalloproteinase
MSC: mesenchymal stem cell
OPG: osteoprotegerin
pAsp: poly-aspartic acid
P2CK: water-soluble two-photon photosensitiser
PEG: polyethylene glycol
PHEX: phosphate-regulating endopeptidase homolog X-linked
PILP: polymer-induced liquid-phase precursor
PIPS: photopolymerization-induced phase separation
PTGS2: prostaglandin-endoperoxide synthase 2
PTH: parathyroid hormone
PUFFS: pulsatile unidirectional fluid flow stimuli
PVA: polyvinyl alcohol
qBEI: quantitative backscattered electron imaging
RANKL: receptor activator of nuclear factor kappa-B ligand
RUNX2: runt-related transcription factor 2
SAED: selected area electron diffraction
SAXS/WAXS: small-angle/wide-angle X-ray scattering
SEM: scanning electron microscopy
SHG: second harmonic generation
SOST: sclerostin
TEM: transmission electron microscopy
TIMA: TESCAN Integrated Mineral Analyzer
TINA: Tool for Image and Network Analysis
TRAP: tartrate-resistant acid phosphatase
VBP: volumetric bioprinting
XRD: X-ray diffraction
β-TCP: beta-tricalcium phosphate
